# Pan-cancer investigation of psoriasis-related BUB1B gene: genetical alteration and oncogenic immunology

**DOI:** 10.1038/s41598-023-33174-3

**Published:** 2023-04-13

**Authors:** Xiaobin Li, Wenwen Wang, Xiaoxia Ding

**Affiliations:** 1 Department of Orthopedic Surgery, Linping Campus, The Second Affiliated Hospital of Zhejiang University School of Medicine, Hangzhou, Zhejiang China; 2 Center for Plastic and Reconstructive Surgery, Department of Dermatology, Zhejiang Provincial People’s Hospital, Affiliated People’s Hospital, Hangzhou Medical College, Hangzhou, Zhejiang China; 3 Department of Dermatology and Venereology, The First Affiliated Hospital of Wenzhou Medical University, Wenzhou, 325000 Zhejiang China

**Keywords:** Cancer, Computational biology and bioinformatics, Immunology

## Abstract

Unknown factors contribute to psoriasis' hyperproliferative, chronic, inflammatory, and arthritic features. Psoriasis patients have been linked to an increased risk of cancer, though the underlying genetics remain unknown. Since our prior research indicated that BUB1B contributes to the development of psoriasis, we designed and carried out this investigation using bioinformatics analysis. Using the TCGA database, we investigated the oncogenic function of BUB1B in 33 tumor types. To sum up, our work sheds light on BUB1B's function in pan-cancer from various perspectives, including its pertinent signaling pathways, mutation locations, and connection to immune cell infiltration. BUB1B was shown to have a non-negligible role in pan-cancer, which is connected to immunology, cancer stemness, and genetic alterations in a variety of cancer types. BUB1B is highly expressed in a variety of cancers and may serve as a prognostic marker. This study is anticipated to offer molecular details on the elevated cancer risk that psoriasis sufferers experience.

## Introduction

Psoriasis (Ps) is a disease with chronic, hyperproliferative, inflammatory, and arthritic characteristics and unclear mechanisms that influences approximately 2–4% of the population^1^. Previous studies have reported that psoriasis sufferers were exposed to higher cancer risk compared with normal individuals, which may be caused by impaired immune surveillance, immunomodulatory therapy, and chronic inflammation^2,3^. More study is still needed to further explanation of the potential processes associated with this elevated risk.

Microarray platforms are becoming more popular as a means to find biomarkers and detect genetic changes in many different diseases^4,5^. Previous research using microarray data has linked several biomarkers and pathways to the emergence of Ps^6–8^. Detailly, natural killer cell-mediated cytotoxicity signaling, TLR4-MyD88-NF-κB signaling pathway, and FCγR-mediated phagocytosis were reported to be correlated with psoriasis progression. The cancer-related genomics in Ps has also been reported, such as CLEC2B^9^ and the IL-17 signaling pathway^10^. However, there is currently a dearth of pertinent pan-cancer analyses.

Previous research suggested that BUB1B plays a role in the development of psoriasis^11^. BUB1B is an essential component of the mitotic checkpoint and is required for proper mitosis^12^. BUB1B deficiency frequently results in aneuploidy and chromosomal instability, which may increase the risk of cancer^13^. Given that, we further carried out a variety of pan-cancer analyses with BUB1B, including expression level analysis, immune cell infiltration, gene mutations, and so on. We also performed enrichment analysis on the BUB1B-associated gene collection to offer a full architecture of the BUB1B genome. With these investigations, we intend to shed light on the molecular basis of psoriasis patients' elevated cancer risk.

## Materials and methods

### Datasets collection

The pan-cancer dataset was gained from the UCSC database (https://xenabrowser.net/): TCGA TARGET GTEx (PANCAN, N = 19,131, G = 60,499)^14^.

### Pan-cancer expression analysis of BUB1B

We extracted the BUB1B [ENSG00000156970] expression data from UCSC (PANCAN, N = 19,131, G = 60,499), and then we transformed each expression value using the log2 function. We investigated the differences in BUB1B expression levels between both normal and cancer samples as well as paired tumors and adjacent normal tissues with R software (statistical packages car and stats) and tested the significance of differences with unpaired Wilcoxon crossover samples. The outcomes were displayed using ggplot2 R package^15^ (https://cran.r-project.org/web/packages/ggplot2/index.html).

### Prognostic analysis

The TCGA prognostic profile was obtained from the UCSC database. All malignancies with fewer than ten samples were eliminated. The relationship between BUB1B expression and prognosis in multiple cancer species was investigated. The survivor R software package was used for proportional risk hypothesis testing (log-rank test) and fitted survival regression. The results of OS (overall survival), DSS (disease-specific survival), DFI (disease-free interval), and PFI (progression-free interval) analysis were presented as Kaplan–Meier curves and forest plots using the survminer and ggplot2 packages. The hazard ratios (HRs) and 95% confidence intervals were calculated using univariate survival analysis.

### Genetic alteration analysis of BUB1B in pan-cancer

The cBioPortal database (http://www.cbioportal.org/), which allows anybody to download, analyze, and view large-scale cancer genomic datasets for a range of malignancies, was used to uncover genetic modifications of BUB1B in pan-cancer. “TCGA Pan-Cancer Atlas Studies” was chosen as the cohort. Next, we typed “BUB1B” into the “Query” module. Alteration sites, types, and numbers for BUB1B could be found in the “cancer type summary” and “mutation” modules. Additionally, using the GDC database (https://portal.gdc.cancer.gov/) and MuTect2 software^16^, we downloaded, processed, and integrated the Simple Nucleotide Variation dataset of all TCGA samples. The structural domain information of the protein was obtained from the R package maftools (version 2.2.10)^17^. The outcome was depicted using a lollipop plot. A chi-square test was also performed to examine the frequency of mutations in different cancer types in two groups that were separated by BUB1B expression level (high or low).

### Correlation of BUB1B expression with immune cell infiltration and immune regulator genes

The infiltration score for each immune cell type was determined based on gene expression for each patient in each tumor using both the xCell^18^ and Timer^19^ methods of the R package IOBR^20^. The expression data for BUB1B and 60 marker genes from the inhibitory (24) and stimulatory (36) immune checkpoint pathways were also examined in each sample^21^. Spearman correlation between BUB1B and an inhibitory as well as a stimulatory immune checkpoint was determined (statistically significant was defined as *p* < 0.05). Recently, a method called ESTIMATE, which computes an immunological score based on certain gene expression patterns of immune cells, has been utilized to forecast the infiltration of non-tumor cells. In this study, an R package called ESTIMATE^22^ (version 1.0.13) was taken to test stromal score, immune score as well as ESTIMATE score based on gene expression for every patient in each tumor type.

### Correlation analysis between BUB1B expression and tumor stemness

We obtained tumor stemness scores including DNAs and RNAs for each tumor from prior studies^23^. After removing cancer species with fewer than 3 samples per tumor type, we ultimately got expression data for 37 cancer species. Then, we intersected BUB1B gene expression data with RNAss or DNAss to calculate the Spearman correlation coefficient. The results were visualized with a lollipop plot using the ggplot2 R package.

### Gene set enrichment analysis

The top 100 BUB1B-correlated genes with the most comparable expression patterns to BUB1B were extracted from the TCGA datasets using the “Similar Gene Detection” module of GEPIA2^24^. Additionally, the STRING tool^25^ (https://string-db.org/) was taken to construct the BUB1B (Homo sapiens) organizational network with the basic parameters as followed: One of the active interaction sources is co-expression. The second active interaction source is evidence. There can be a maximum of 50 interactors. Medium confidence is the lowest required interaction score (0.40). GAGE^26^ was used to analyze the Kyoto Encyclopedia of Genes and Genomes (KEGG) pathway data, and the ggplot2 R package was used to visualize the results. The KEGG database (http://www.genome.jp/kegg) provides annotation of the genes and pathways^27^. And *p* < 0.05 is the significance cutoff.

## Results

### Pan-cancer expression analysis of BUB1B

**Figure 1:**
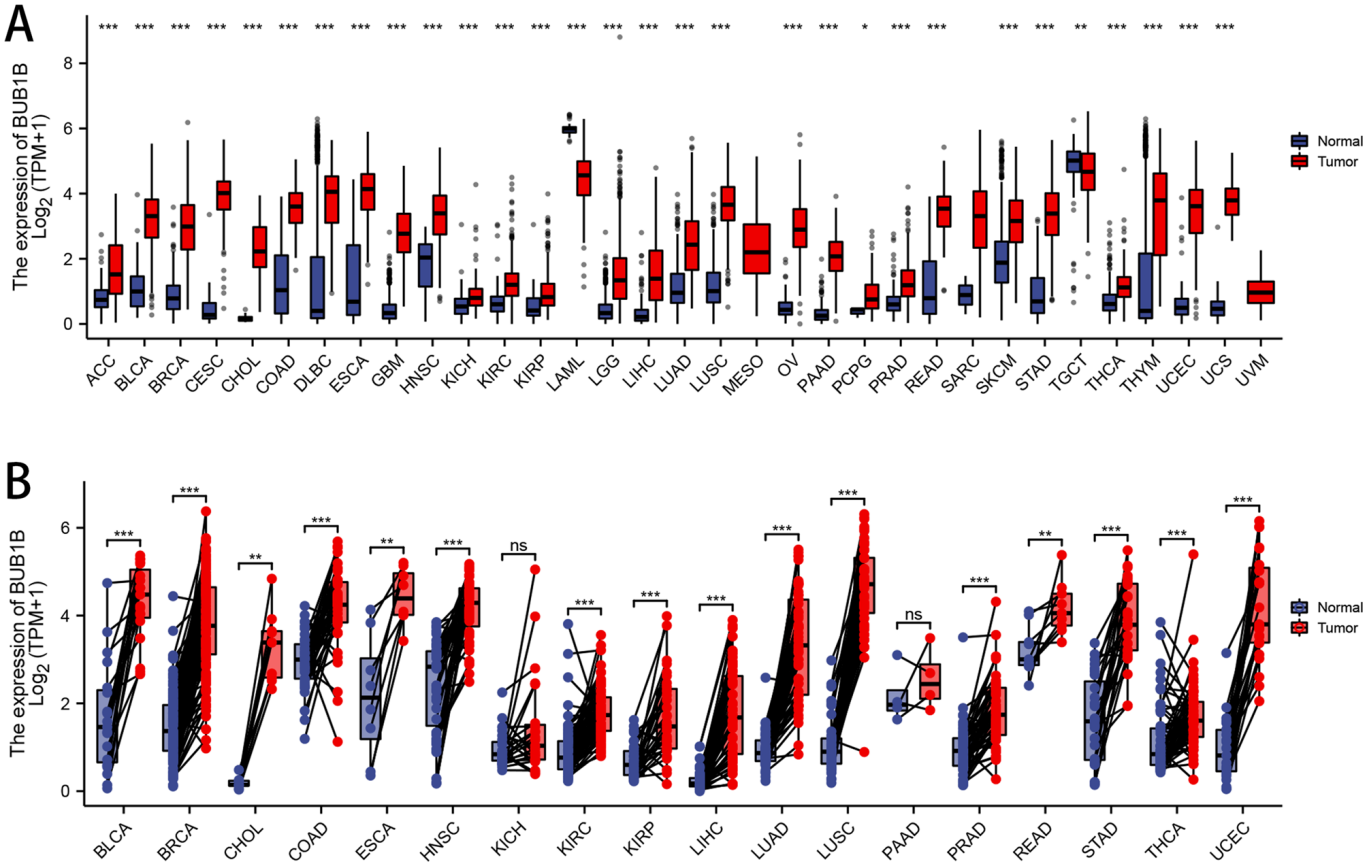
Expression patterns of BUB1B in pan-cancer. (**A**) For unpaired normal and tumor samples; (**B**) For paired tumor and normal tissues.

We found substantial upregulations of BUB1B in 33 tumors, as shown in Fig. 1A, including GBM, GBMLGG, LGG, UCEC, BRCA, WT, CHOL, CESC, ACC, LUAD, PCPG, ESCA, KICH, STES, LAML, KIRP, ALL, KIPAN, UCS, PAAD, COAD, OV, READ, COADREAD, THCA, BLCA, PRAD, SKCM, LIHC, STAD, LUSC, KIRC, and HNSC; and significant deregulation in 1 tumor type (THYM). BUB1B was found to be highly expressed in 18 different cancers in the TCGA pan-cancer tumor and normal tissues (Fig. 1B), including BLCA, BRCA, COAD, CHOL, ESAD, LUSC, STAD, THCA, ESCA, HNSC, KIRP, KIRC, PRAD, LIHC, UCEC, READ, OSCC, and LUAD.

### Prognostic significance of BUB1B

**Figure 2:**
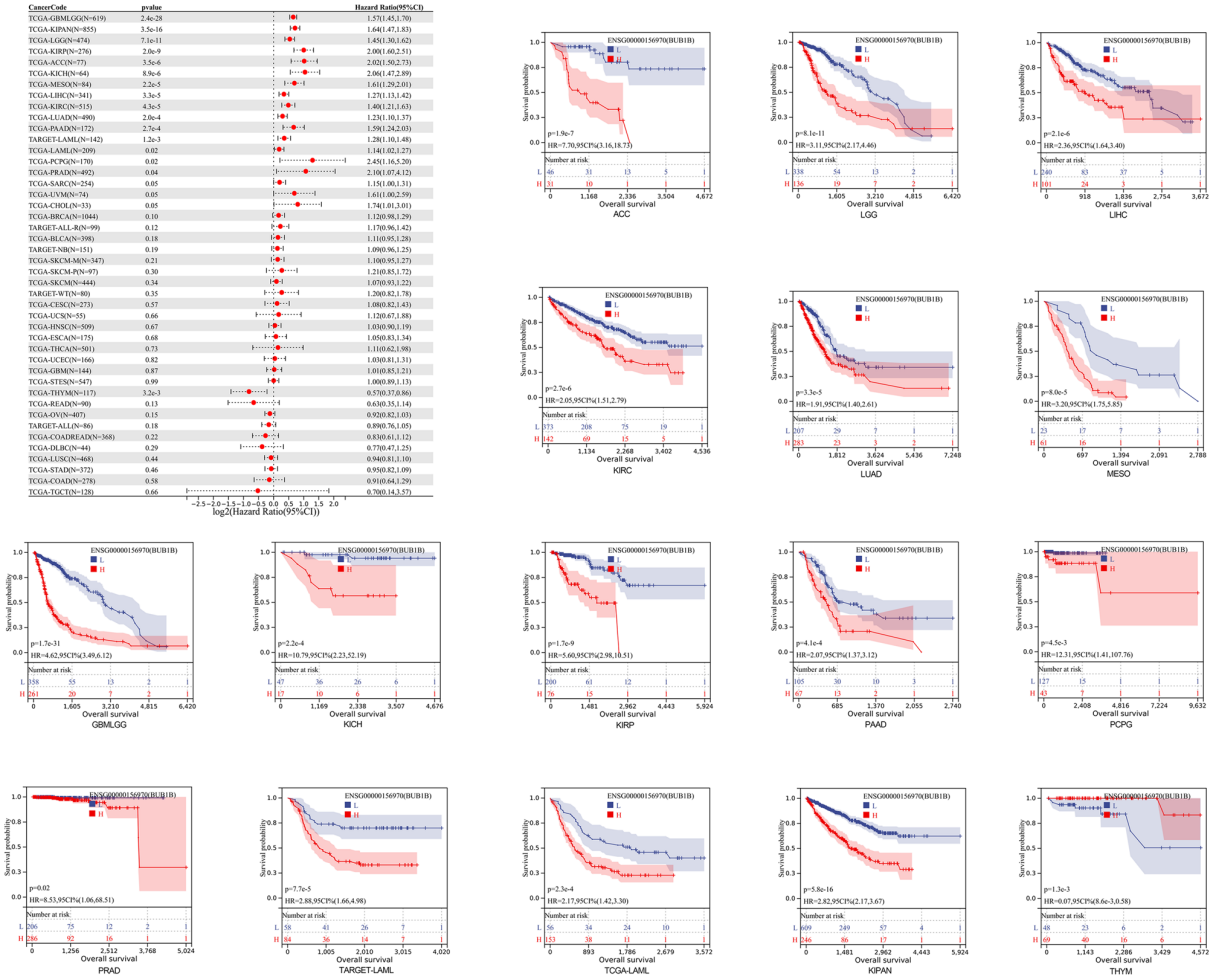
The relationship between BUB1B expression and OS in pan-cancer.

**Figure 3:**
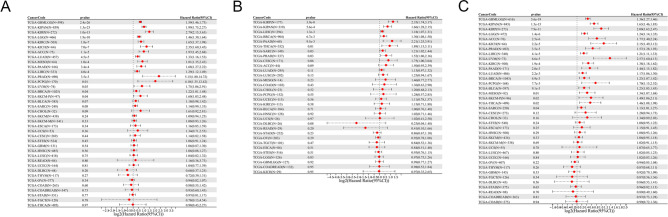
The relationship between the expression of BUB1B and DSS (**A**), DFI (**B**), and PF1 (**C**) in pan-cancer.

We discovered that poor OS in 14 tumor types was significantly correlated with increased BUB1B expression (KIPAN, GBMLGG, LGG, KIRP, ACC, KICH, MESO, LIHC, KIRC, LUAD, PAAD, LAML, PCPG, PRAD). The results yielded from KM curves were similar to the previous findings (Fig. 2). The relationship between BUB1B expression level and DSS, DFI, as well as PFI was also calculated in each cancer type. As shown in Fig. 3, upregulated expression of BUB1B was remarkably correlated with poor DSS (A), DFI (B), and PF1 (C) in 16 tumor types (GBMLGG, KIPAN, KIRP, LGG, KIRC, KICH, ACC, LUAD, MESO, PAAD, LIHC, PRAD, PCPG, UVM, BRCA, SKCM-P).

### Genetic alteration of BUB1B

**Figure 4:**
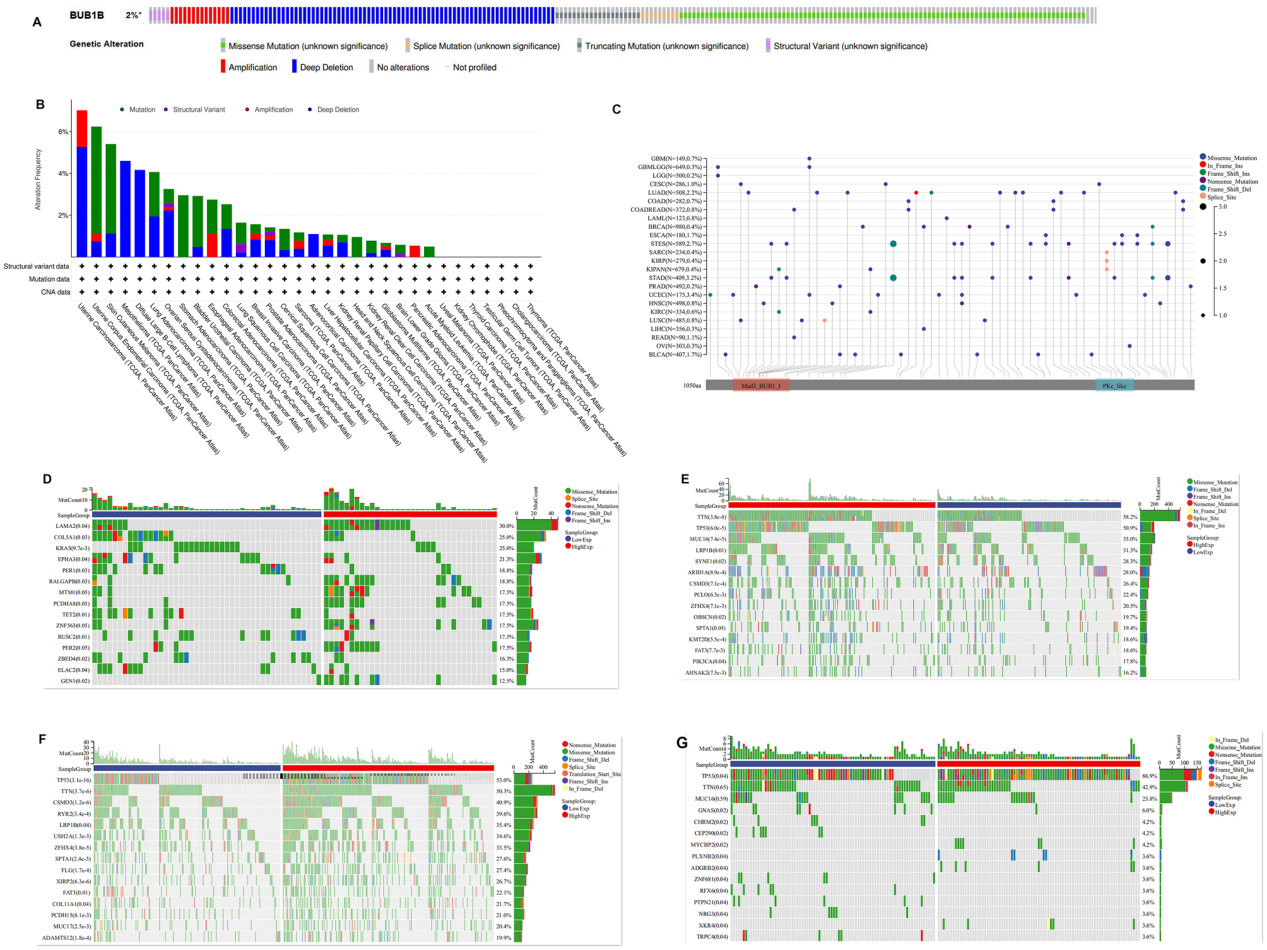
Genetic alteration of BUB1B in pan-cancer. (**A**), (**B**) The genetic alteration ratios, types, and frequency of BUB1B in pan-cancer. (**C**) The mutational landscape of BUB1B in pan-cancer. (**D**)–(**G**): Genetic alteration differences in UCEC, STAD, LUAD, and ESCA with different expression levels of BUB1B.

**Figure 5:**
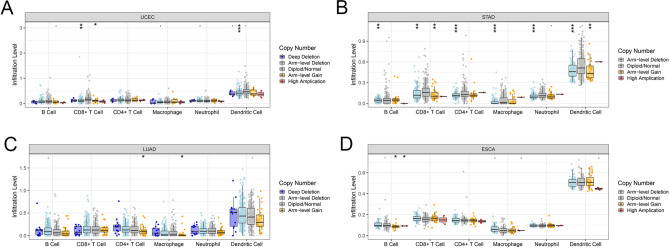
The correlation between the SCNAs of BUB1B and immune infiltration in UCEC, LUAD, STAD, and ESCA from the TIMER database.

The results showed that the alteration rate of BUB1B in pan-cancer was 2% of quired samples (Fig. 4A). Genetic alteration types and frequency of BUB1B showed differences in pan-cancer (Fig. 4B). The top 3 alteration frequencies were 7.02% in Uterine Carcinosarcoma, 6.24% in Uterine Corpus Endometrial Carcinoma, and 4.6% in Skin Cutaneous Melanoma, respectively. As shown in Fig. 4C, BUB1B was widely mutated across multiple cancer species, with the top 5 high-frequency mutations being Stomach and Esophageal carcinoma (STES), Uterine Corpus Endometrial Carcinoma (UCEC), Stomach adenocarcinoma (STAD), Esophageal carcinoma (ESCA), and Lung adenocarcinoma (LUAD) at 3.40%, 3.20%, 2.70%, 2.20%, and 1.70%. We used chi-square testing to assess differences in the frequency of gene mutations in the aforementioned 4 tumor types (UCEC, STAD, LUAD, ESCA), and the results were shown in Fig. 4D–G. TP53 and TTN were the top 2 mutated genes in STAD, LUAD, and ESCA. TTN mutation did not demonstrate a statistically significant difference between various expressed BUB1B groups in ESCA. TP53 mutations were more frequent in high-expressed BUB1B groups in STAD, LUAD, and ESCA. Additionally, the SCNA module of the TIMER was used to investigate the relationship between the SCNAs of BUB1B and the immune infiltration pattern. The results showed that SCNAs of BUB1B reduced immune cell enrichment in the aforementioned cancer types (Fig. 5), especially in STAD, indicating a clear link between BUB1B genetic alteration and immune cell infiltration enrichment.

### Relationship of BUB1B expression and immunological environment in Pan-cancer

**Figure 6:**
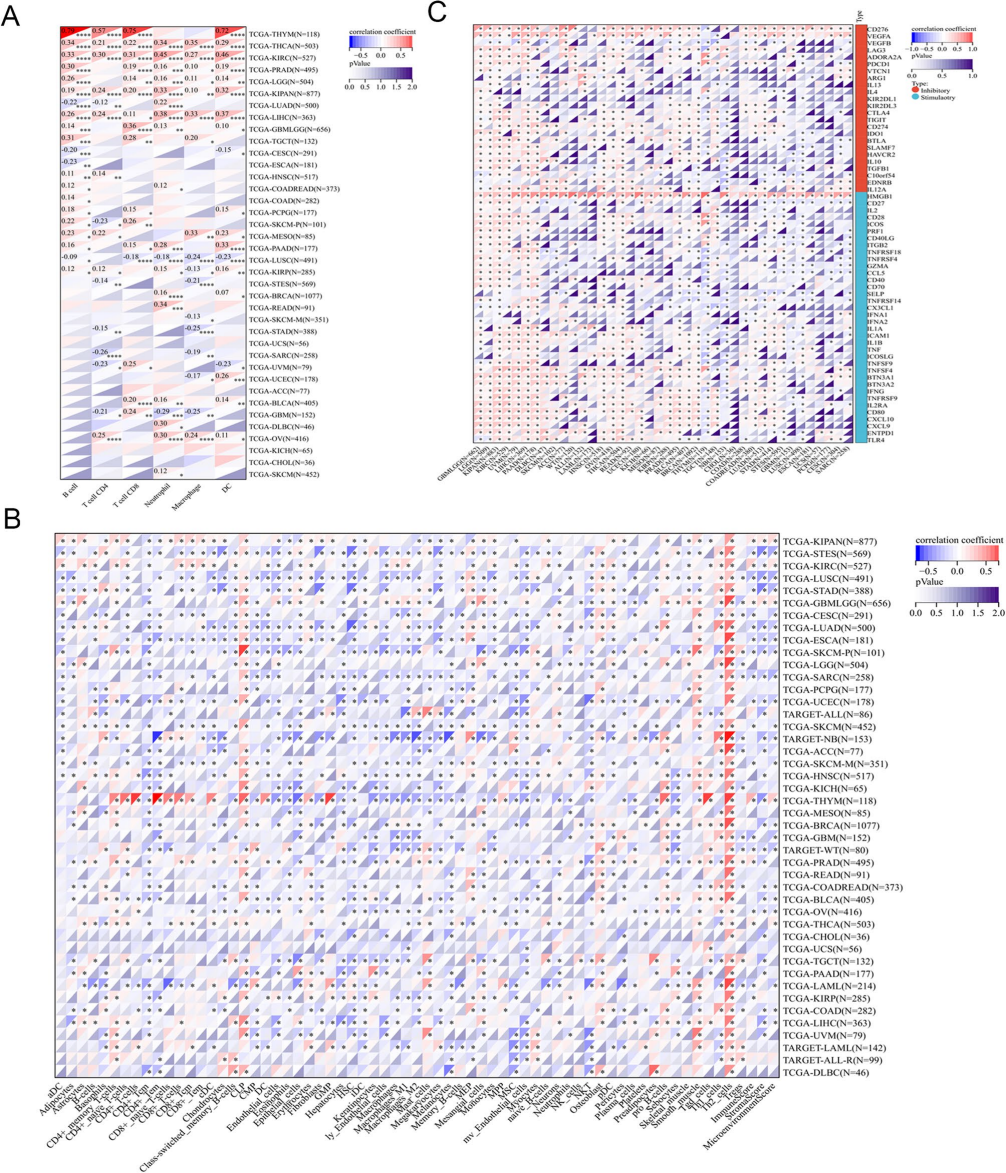
BUB1B expression and immunological correlation in Pan-cancer. (**A**) TIMER method; (**B**) xCell method; (**C**) association of BUB1B expression and immune checkpoints in pan-cancer.

Using the TIMER approach (Fig. 6A), we found that the expression of BUB1B was substantially associated with the quantity of invading immune cells: CD4+ T cells in 16 species, neutrophils in 19 species, B cells in 21 types, macrophages in 17 types, CD8+ T cells in 16 types, and DCs in 19 types. We discovered that the majority of the 38 immune cell subtypes are significantly linked with BUB1B expression in various tumor types using the deconvo xCell method. BUB1B expression was most strongly correlated with Th2 cells in various malignancies (Fig. 6B). Cancer patients' prognosis is affected by immunosurveillance, and tumors manipulate immunological checkpoints to elude immune responses. According to our results, the majority of immunomodulators found in LUSC, THYM, and NB were negatively associated with BUB1B expression. However, in GBMLGG, KIPAN, LIHC, and PAAD, the expression of BUB1B was positively linked with the majority of immune inhibitors, and immunostimulators (Fig. 6C).

### Correlation analysis with the immune score using the ESTIMATE algorithm

**Figure 7:**
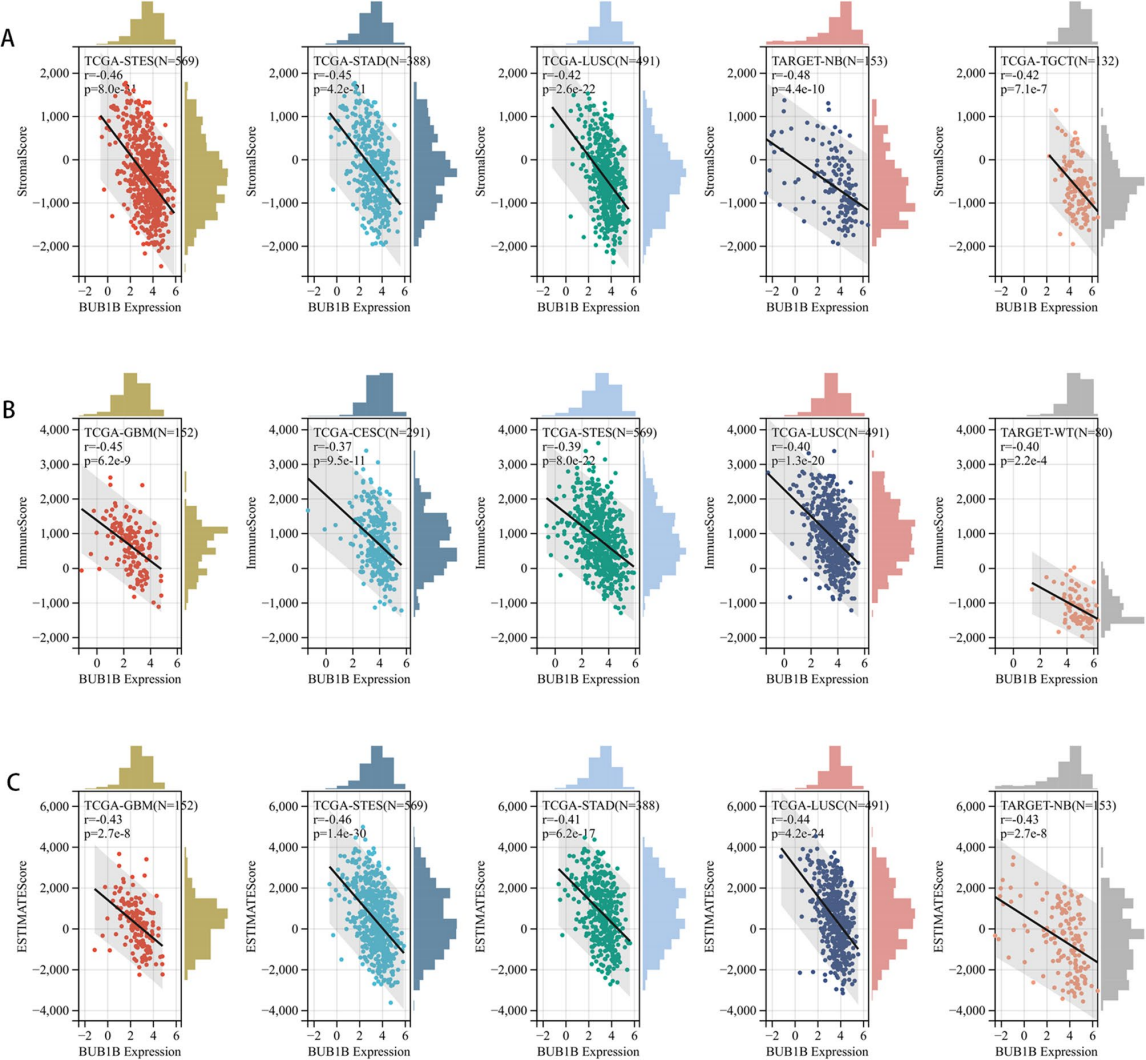
Association of BUB1B expression and immune microenvironment scores in pan-cancer. (**A**) Stromal score; (**B**) Immune score; (**C**) ESTIMATE score.

We calculated immune infiltration scores for a total of 10,178 tumor samples from 44 tumor types, and Spearman’s correlation coefficient was determined between the levels of immune infiltration and BUB1B expression using corr. test function of the R package psychology (version 2.1.6). Among them, the BUB1B expression pattern was associated with immune infiltration in 22 types of tumors, of which 3 were significantly positive (GBMLGG, KIPAN, KIRC) and 19 negatives (GBM, UCEC, CESC, BRCA, ESCA, LUAD, STES, SARC, COAD, READ, STAD, HNSC, LUSC, TARGET-WT, SKCM, OV, BLCA, PCPG, ACC). Given that, we assume that high expression of BUB1B decreased both the immune cell and stromal cell infiltration but increased the number of tumor cells, which leads to a bad prognosis. We ranked all of the engaged cancer kinds based on the absolute value of r in the three types of scores mentioned above, and the top five cancer types are presented in Fig. 7.

### Correlation between BUB1B expression and tumor stemness

**Figure 8:**
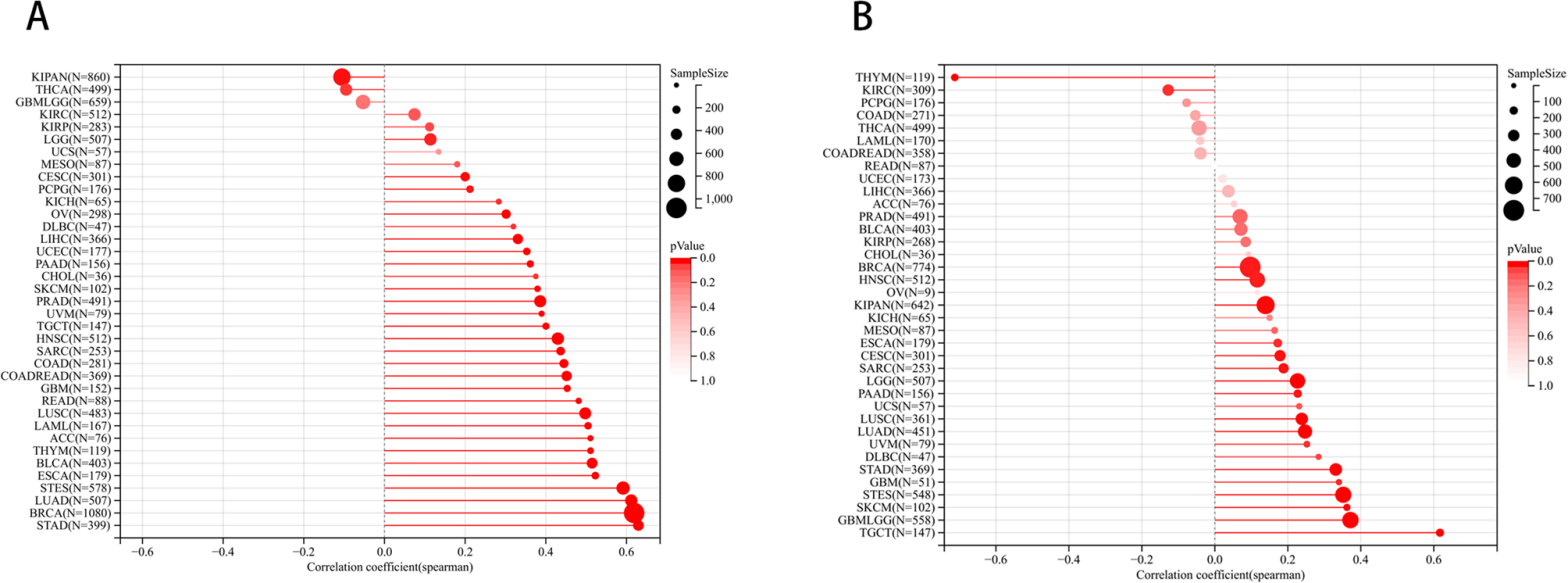
Correlation between BUB1B expression and tumor stemness. (**A**) Association of BUB1B and RNAss in pan-cancer; (**B**) Association of BUB1B and DNAss in pan-cancer.

As cancer progresses, tumor cells may shed their differentiated phenotype and acquire progenitor and stem cell characteristics. Tumorigenic stemness can be assessed using RNA-derived mRNA expression (RNAss) and DNA-derived DNA methylation patterns (DNAss)^23^. The link between BUB1B expression and tumor stemness, as determined by RNAss and DNAss, was examined in the current study. The Spearman correlation for each tumor was evaluated, and BUB1B interacts with RNAss (Fig. 8A) and DNAss (Fig. 8B) to varied degrees. In particular, we discovered that 19 cancers (17 positives) had substantial correlations in DNAss, while 32 tumors (30 positives) had strong correlations in RNAss. Notably, BUB1B showed a positive association with DNA and RNA for several different forms of cancer types but showed a negative correlation with RNA for THYM. These contradictory findings imply that RNAss and DNAss may be able to distinguish between various malignant cell populations with varying characteristics or levels of stemness in various cancers.

### BUB1B-related gene enrichment analysis

**Figure 9:**
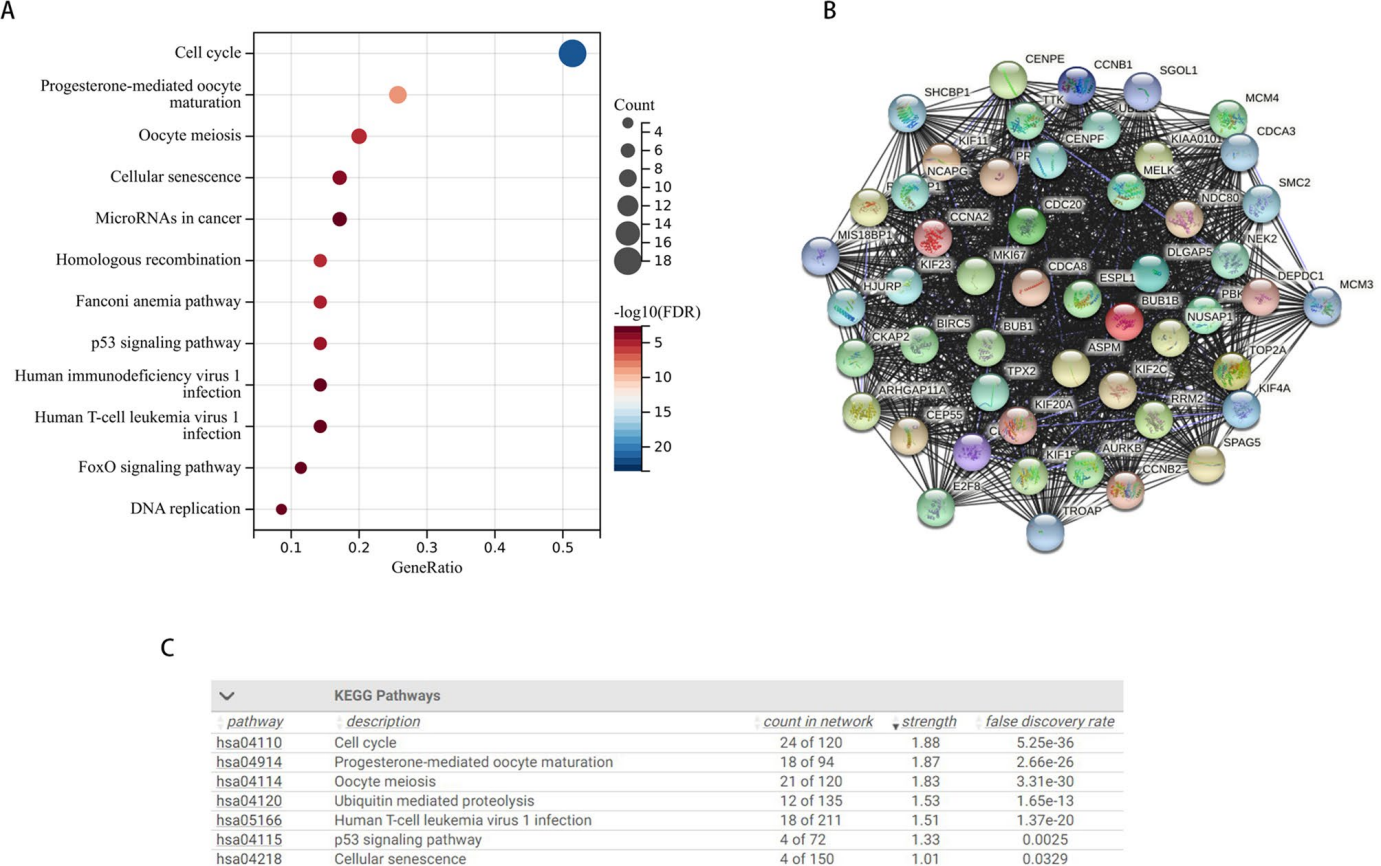
Gene sets enrichment analysis of BUB1B. (**A**) KEGG enrichment analysis of gene set from GEPIA. (**B**) PPI network of BUB1B-related genes from STRING online tool. (**C**) KEGG enrichment analysis of gene set from STRING.

To examine the functional mechanism of BUB1B in carcinogenesis, we gained the top 100 genes with similar expression patterns comparable to BUB1B from GEPIA2. The KEGG enrichment analysis revealed that the aforementioned 100 genes were strongly linked to the cell cycle or cellular senescence (Fig. 9A). The STRING tool was then used to gather 50 genes that co-expressed with BUB1B in order to validate the findings. The 50 genes were closely correlated, as seen in Fig. 9B, the genes were likewise involved in the cell cycle and cellular senescence (Fig. 9C). These findings lead us to hypothesize that BUB1B, through regulating the cell cycle and inducing cellular senescence, may contribute to tumorigenesis in malignancies.

## Discussion

Several studies have been conducted on the high cancer risk in psoriasis patients, with the emphasis mostly on the effect of lifestyle (e.g., smoking and alcohol use) or treatment (e.g., PUVA or UVB irradiation and biologics)^2,28,29^. There are several factors that can contribute to cancer, but the most prevalent are exogenous factors and endogenous factors^30^. With the advent of genetic testing and the era of targeted therapy, molecular signatures have become increasingly important^31^. However, there is currently a scarcity of studies on the molecular basis. According to our prior research, BUB1B might be a gene that bridges the gap between psoriasis and cancer development^11^. Based on this, we sought to investigate the role of BUB1B in pan-cancer and provide potential molecular mechanisms for psoriasis and the concomitant high risk of cancer.

BUB1B is a critical component of the mitotic checkpoint, which is required for normal mitosis^32^. While BUB1B over-expression is reported in many different cancers, including breast cancer^33^, ECC^34^, PCa^35^, and so on, there has not been a comprehensive pan-cancer analysis of BUB1B’ status. This study found that BUB1B was significantly upregulated in multiple tumors across both paired (18 tumor types) and unpaired (33 tumor types) sample analyses, which is consistent with the literature. On the basis of these results, we investigated the implications of differential BUB1B expression on prognosis, tumor immunity, and gene mutation in pan-cancer. Poor OS in 14 tumor types was revealed to be substantially linked with higher BUB1B expression using Cox and KM curve analysis (KIPAN, GBMLGG, LGG, KIRP, ACC, KICH, MESO, LIHC, KIRC, LUAD, PAAD, LAML, PCPG, PRAD). Similar results were found in previous studies that high BUB1B expression was associated with poor prognosis in the three types mentioned above, suggesting that BUB1B is a viable prognostic factor for certain malignancies. A genetic alteration is an essential event in cancer development and progression. Moreover, the differences in the genetic molecular features of patients, such as DNA methylation, mutation, and copy number alterations (CNA), have been shown to be associated with clinical responses to anti-cancer drugs^36,37^. Given this, we investigated the genetic alteration of BUB1B in pan-cancer further. Stomach and Esophageal Carcinoma (STES), Uterine Corpus Endometrial Carcinoma (UCEC), Stomach Adenocarcinoma (STAD), Esophageal Carcinoma (ESCA), and Lung Adenocarcinoma (LUAD) had the highest mutation rates, with frequencies of 3.4%, 3.2%, 2.7%, 2.2%, and 1.7%, respectively. Somatic copy number alteration (SCNA) is an important form of somatic genetic alteration in cancer^38^. We then examined the association between the SCNAs of the BUB1B gene and immune infiltration in the aforementioned cancer types using the SCNA module of TIMER. The results showed that different SCNAs of BUB1B could decrease immune cell infiltration in aforementioned cancer types and have the greatest effect on STAD, suggesting that the genetic alteration of BUB1B is closely associated with immune cell infiltration.

However, the function of BUB1B and its impact on the tumor immune milieu have received little attention so far. Thus, we further investigated the relationship between BUB1B expression and tumor immune environment in pan-cancer. Using the TIMER method, it was revealed that BUB1B is significantly associated with B cell, CD8+ T cell, CD4+ T cell, macrophage, neutrophil, and DC infiltration levels in different cancers. Using the deconvo xCell approach, BUB1B expression was also found to be significantly associated with the majority of the 38 immune cell subtypes in diverse tumor types, especially Th2 cell (positive correlation), which demonstrate pro-cancer function via the secretion of interleukin-4 (IL-4), interleukin-6 (IL-6), interleukin-10 (IL-10), and transforming growth factor-β (TGF-β)^39^. Traditionally, psoriasis has been thought to be a T helper type 1 (Th1)-dominated skin condition, but emerging research indicates that the Th1-Th2-Th17 balance is likely a crucial functional and genetic determinant of psoriasis^40–42^. The relationship between BUB1B and Th2 cells hasn’t attracted attention yet, which may be a new research avenue. Utilizing the ESTIMATE algorithm, we discovered that high BUB1B expression lowered immune cell and stromal cell infiltration while increasing tumor cell quantity in 19 cancer types (GBM, UCEC, CESC, BRCA, ESCA, LUAD, STES, SARC, COAD, READ, STAD, HNSC, LUSC, TARGET-WT, SKCM, OV, BLCA, PCPG, ACC). The aforementioned impacts were most pronounced in STES, STAD, and LUSC. The present findings show a close relationship between BUB1B and the tumor immunological environment, implying that BUB1B could be a promising research and therapeutic target, however, further validation trials are required.

Growing evidence demonstrates that cancer stemness and immune evasion play a critical role in tumor development, progression, and metastasis^43^. Interestingly, a strong association between the expression of BUB1B and the majority of immunosuppressive as well as immunostimulatory molecules in various tumor types was also looked at in this study. Furthermore, in different cancer types, BUB1B demonstrated varying levels of connection with RNAss and DNAss (mainly positive association), implying that BUB1B may contribute to tumor progression by boosting cancer stemness. As a result, we hypothesize that increased BUB1B expression may promote cancer formation and potentially treatment resistance by boosting tumor stemness and assisting in immune surveillance evasion. To further uncover the possible signaling pathways involved in BUB1B in pan-cancer, we first obtained the top one hundred most similar gene sets to BUB1B using GEPIA and performed enrichment analysis on this gene set. The results showed that “cell cycle” was the most prominent, but “MicroRNAs in cancer”, “Homologous recombination”, “p53 signaling pathway”, “FoxO signaling pathway” and “DNA replication” was also not negligible. This suggests BUB1B may contribute to cancer malignancy and progression through multiple mechanisms.

To summarize, our findings shed light on BUB1B’s role in pan-cancer from a variety of angles, including pan-cancer expression, prognostic roles, genetic alterations, tumor stemness, and the possible correlation with tumor immune microenvironment, providing a theoretical foundation for the possibility of BUB1B as a pan-cancer marker. Yet, this study, which spanned various databases, had certain drawbacks. We performed a bioinformatic investigation of BUB1B, which made determining the value of clinical transformation challenging. Furthermore, because all of the databases' resources were tissue-derived, these findings cannot be confirmed with in vitro/in vivo models. Ultimately, further research into the mechanisms involved in carcinogenesis is needed.

## Data Availability

The datasets generated and analyzed during the current study are available in the UCSC database: UCSC Xena (xenabrowser.net).
